# Excision of a large trichilemmal carcinoma of the back with staged reconstruction using biodegradable temporizing matrix (BTM): A case report

**DOI:** 10.1016/j.jpra.2024.05.010

**Published:** 2024-05-31

**Authors:** Niamh Gorman, Susan McCrossan, Jason Kelly

**Affiliations:** Department of Plastic Surgery, Cork University Hospital, Wilton, Cork, Ireland

**Keywords:** Trichilemmal carcinoma, Back, Biodegradable temporizing matrix (BTM)

## Abstract

Trichilemmal carcinomas (TC) are rare skin malignancies that arise from the external root sheet of a hair follicle. Their incidence increases with advanced age and they most commonly occur on sun exposed skin or areas of significant hair growth. They vary significantly in size and appearance. Surgical excision is the most common treatment option. We report the case of a large trichilemmal carcinoma of the back occurring in a woman with poorly controlled diabetes mellitus. The lesion was excised resulting in a very large defect, which was reconstructed in a staged process using biodegradable temporising matrix (BTM) and split-skin grafting. There was 95 % graft take at first graft check and the wound was fully healed at 6 weeks post grafting. BTM, already an established adjunct in the reconstruction of burns, degloving injuries and soft tissue infections, provided an enhanced aesthetic outcome and successful wound healing in this complex skin lesion excision.

## Introduction

Trichilemmal carcinoma (TC) is a rare cutaneous neoplasm that originates from the external root sheath of a hair follicle.^1^ TCs share characteristics of some more common cutaneous lesions such as squamous cell (SCC) and basal cell carcinoma (BCC). Whilst they are often asymptomatic and rarely metastasise, TC's can be locally invasive.^1^

Surgical excision with primary closure is the preferred treatment. Split-skin grafting (SSG) and full-thickness grafting (FTG) have been documented as methods for wound closure as well as local and free-flap reconstruction of larger defects.^1^ BTM is a synthetic material that promotes growth of a neodermis, priming the wound bed in advance of SSG.^2^ It has been used in reconstruction post excision of large skin lesions, however, there are no documented cases of BTM use for reconstruction post excision of a TC.

We report the case of a large TC of the back. The lesion was excised, and reconstruction was performed in a two-step process with delayed split skin grafting following BTM application.

## Case report

**Figure 1:**
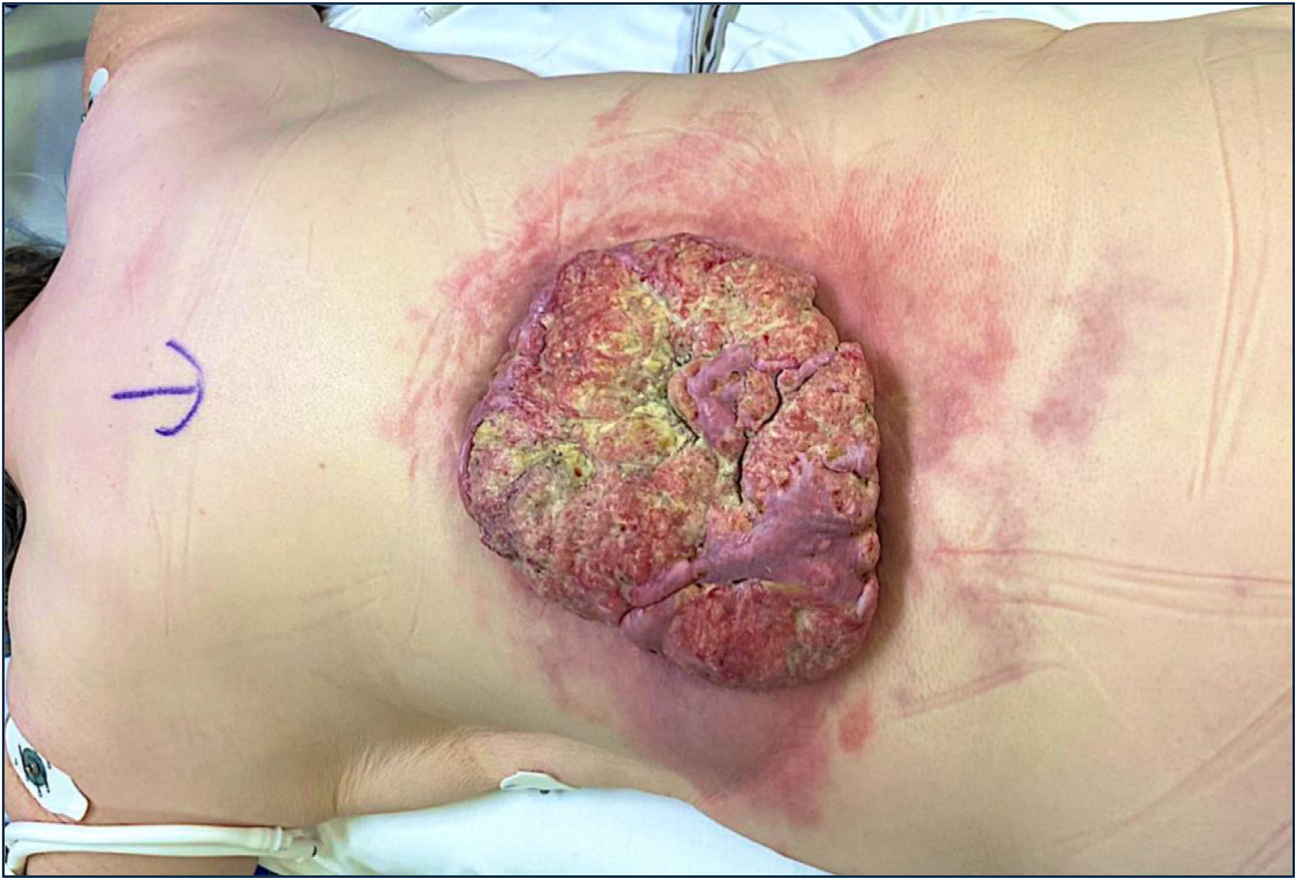
Lesion pre-excision, superior view.

A 58-year-old woman presented with a large lesion on her back which had been evolving for many years. Her background was notable for poorly controlled Type 2 diabetes mellitus. On examination, there was a large exophytic lesion on the thoracolumbar area of her torso (Figure 1). The lesion covered approximately half of her posterior torso, an estimated 8 % TBSA when erythematous skin changes that were part of the disease process were considered.

An excisional biopsy identified a focus of poorly differentiated invasive carcinoma at the deep aspect with telangiectatic blood vessels within the superficial and deep dermis. There was associated fibrosis and chronic inflammation. A second punch biopsy showed marked cellular atypia with a foci of pilar differentiation consistent with trichilemmal carcinoma. The lesion was hot to touch and bled profusely at biopsy. Computed Tomography (CT) showed a large mass extending 16 cm craniocaudally, 15 cm transversely and 5 cm in depth. It overlay T11-L4 vertebrae in the midline with an irregular, ulcerative periphery and small superficial erosions on the spinous processes of L1-L3. There was bilateral axillary lymphadenopathy.

She was referred to the pre-operative assessment clinic at which time her HbA1c was 117. She was urgently reviewed by Endocrinology who recommended commencing dapagliflozin and semaglutide. However, initiation of this treatment was delayed given the increased risk of aspiration under anaesthesia secondary to delayed gastric emptying in semaglutide use.^3^

The lesion was excised to the level of the spinous processes centrally and subfascial level peripherally. A gross margin of 2 cm was obtained intra-operatively. Total excision size was 30 × 27.5 × 3.6 cm, including a mass size of 16.2 × 14.5 × 1.5 cm on the surface which was firm and tan in colour, and a halo of erythema ranging from 3.5 – 8 cm. The greatest depth of the lesion was 3 mm from the base to the deep margin, with fibrosis extending to the deep margin. There were small loculated cystic areas, largest of which was 1.8 cm. The paraspinous muscles were carefully excised, including the periosteum of the spinous processes. The deep fascia was intact.

Biodegradable temporising matrix (BTM) was applied to the wound and inset with multiple clips to ensure adherence and create micro pockets. A VAC was applied with Acticoat 7 as an interface. Negative margins were confirmed on histology and biopsy of left axilla identified no metastasis. A PET scan excluded distant metastasis.

**Figure 2:**
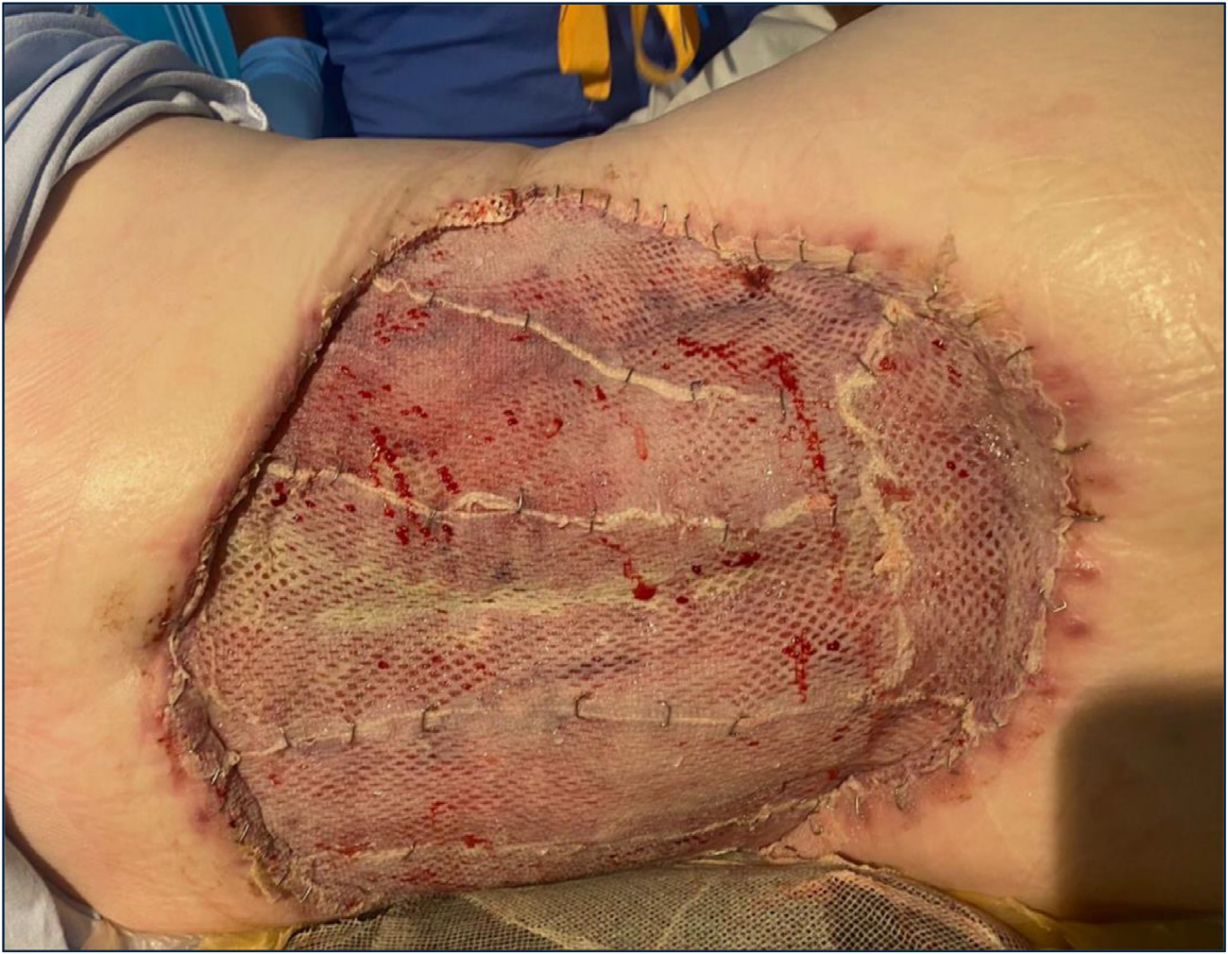
Wound review, day 5 post SSG.

There were no wound complications at 6 weeks post-op with weekly VAC changes. She underwent SSG using a hand fenestrated 1:1.5 mesh secured with skin clips and Artiss, followed by VAC application. There was 95 % graft take at first graft check at Day 5 post grafting (Figure 2).

**Figure 3:**
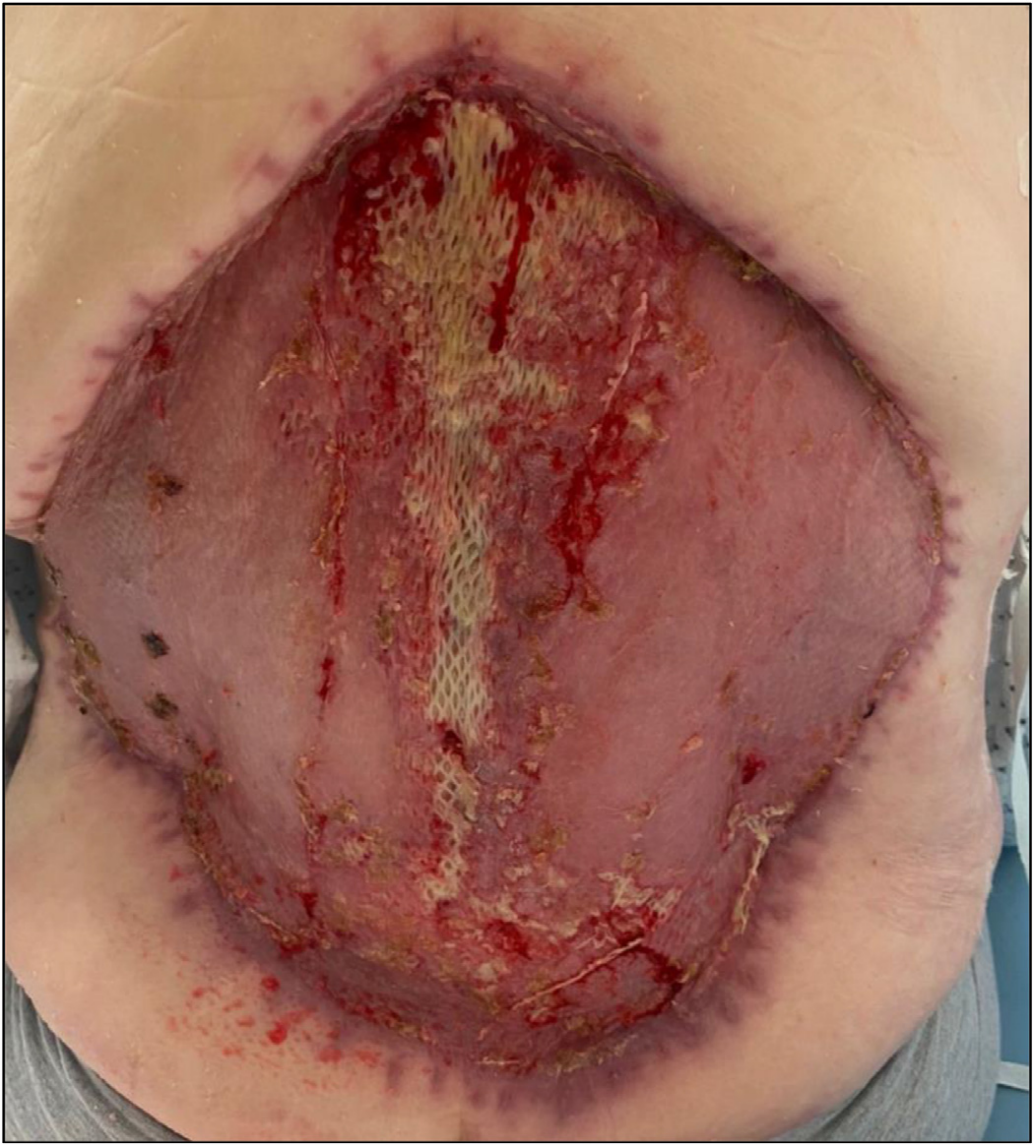
Wound review, 4 weeks post SSG.

**Figure 4:**
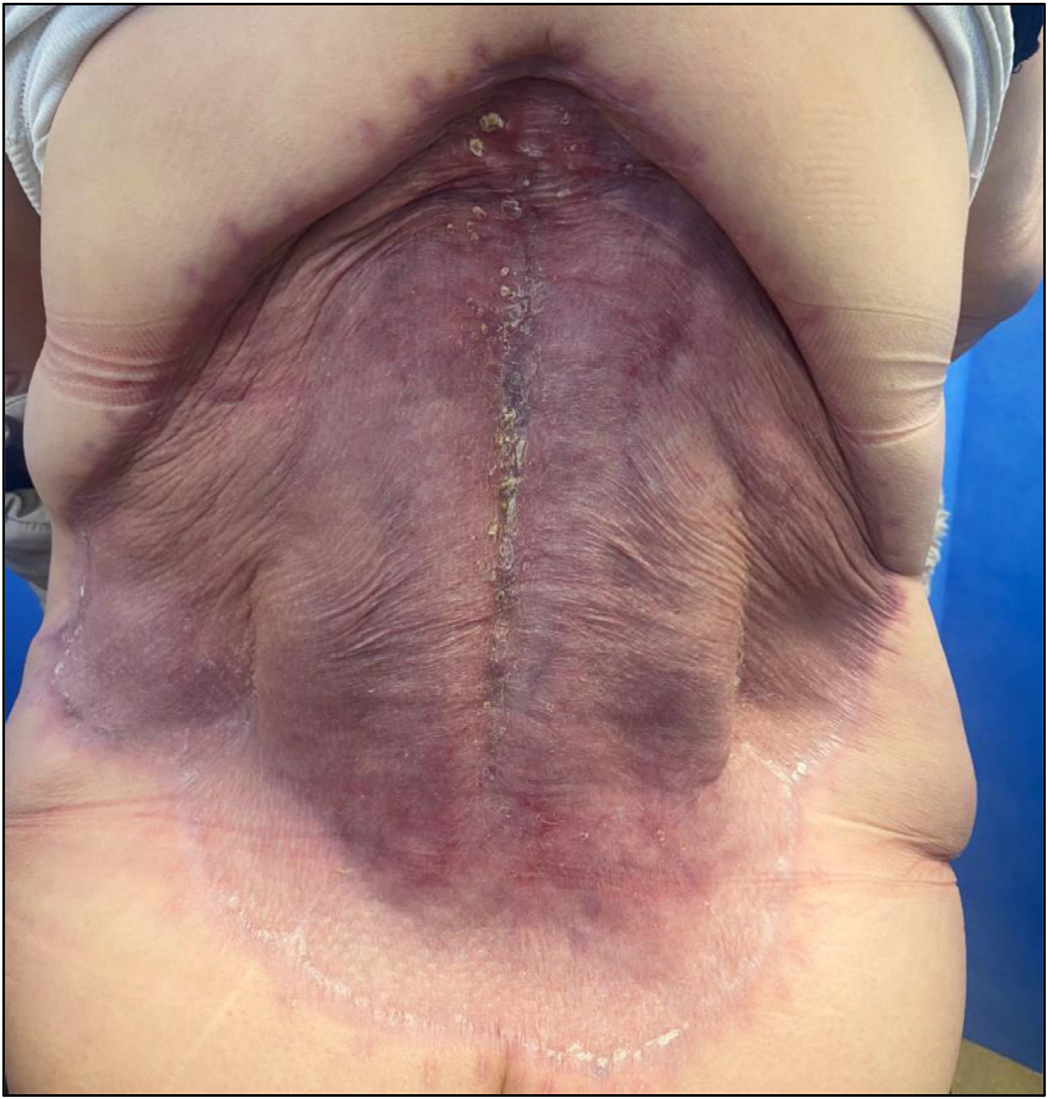
Wound review, 6 months post SSG.

The central portion was slower to take but the wound was fully healed at follow-up at 6 weeks (Figure 3). There was no obvious meshed appearance of the wound that would be expected with split-skin grafting without BTM application (Figure 4).

## Discussion

Trichilemmal carcinoma (TC) is a rare cutaneous tumour that originates from the external root sheath of a hair follicle.^1^ They commonly occur on sun exposed skin, areas of thick hair growth and incidence increases with advanced age.^1^ The above case is atypical as, in addition to the sheer size of the lesion, it was not consistent with the most common locations for occurrence, and our patient was young in comparison to the average age for females (71 years).^1^

TC was first described by Headington in 1976.^6^ Phenotypically, TC's vary significantly in size, location, and morphology.^1^^,^^5^ Eighty four percent of cases occur on the head and neck. Diameter ranged from 0.3 – 37 cm. Only one previously reported case was on the back.^1^ The variability in presentation contributes to the confusion surrounding TC's. Yet, there is an absence of comprehensive research. Therefore, they can easily be misdiagnosed, and histology is required for definitive diagnosis.^1^

In addition to age and sun exposure, several other risk factors have been reported for developing TC. Caucasians and males had an increased incidence of TC.^1^ Pre-existing scars, solid organ transplantation and previous ionising radiation were also identified as risk factors.^7^ Metastasis of trichilemmal carcinoma is rare but has been documented and largely occurred in immunosuppressed individuals. Metastasis is typically via the lymphatics.^1^

TCs share characteristics of some more common cutaneous lesions such as SCC and BCC.^1^ However, they are histologically distinct.^5^ TCs are characterised by trichilemmal keratinization with an absence of a granular layer between the stratum spinosum and stratum corneum. Whilst there are no definite diagnostic criteria, the following features are widely accepted as diagnostic: PAS positive glycogen within the cells, peripheral palisading of clear cells around a central polygonal cell, a positive D-PAS basement membrane, lobular organisation, and presence of a pre-existing trichilemmoma. Cells display a high grade of atypia with a high mitotic index.^4^^,^^5^

Treatment is ordinarily by wide local excision with clear margins.^1^ Radiotherapy is the preferred method where the lesion has been unresectable.^7^ Adjuvant radiotherapy had also been used where adequate margins could not be achieved, with variable outcomes. Combined chemotherapy and radiotherapy are used in the case of metastatic disease and has shown partial response.^1^^,^^7^

BTM has been mostly used in burns, degloving injuries and post soft tissue infection.^2^ BTM yielded favourable results and provides a reconstructive option to patients with large ‘ungraftable’ defects.^8^ It has also been used for reconstruction of large cutaneous tumours.^2^ SSG and FTSG have been previously used post TC excision, in addition to and free flap reconstruction.^1^^,^^9^ In this case, BTM was used successfully for reconstruction of the sizeable defect by optimising graft take enhancing aesthetic outcome. This has not been previously described for TCs.

Prognosis is good and recurrence is rare.^1^ Adequate surgical margins, extent of lymphatic metastasis, history of concurrent carcinoma, diameter, and length of time the lesion has been present are major contributors to recurrence.^1^ Regular follow up is required. Current recommendation are four monthly for two years then six monthly, as per the NICE SCC Guidelines.

## Conclusion

TC is a rare adnexal neoplasm that is grossly similar to other skin lesions but is microscopically distinct. Surgical excision is the treatment of choice and regular follow up is recommended for surveillance for recurrence. Where reconstruction is required, BTM provides favourable aesthetic outcomes as part of a staged reconstruction process.

## Declaration of competing interest

I can confirm that there were no conflicts of interest in the process of completing this work.
